# Roles of Exosomes Derived From Immune Cells in Cardiovascular Diseases

**DOI:** 10.3389/fimmu.2019.00648

**Published:** 2019-03-29

**Authors:** Runda Wu, Wei Gao, Kang Yao, Junbo Ge

**Affiliations:** Department of Cardiology, Zhongshan Hospital, Fudan University, Shanghai Institute of Cardiovascular Diseases, Shanghai, China

**Keywords:** cardiovascular disease, exosome, immune cell, inflammation, biomarker, therapy

## Abstract

Therapies aimed at minimizing adverse remodeling in cardiovascular diseases on a molecular and cellular basis are urgently needed. Exosomes are nanosized lipid vesicles released from various cells that are able to mediate intercellular signaling and communication via their cargos. It has been increasingly demonstrated that exosomes from cardiomyocytes or stem/progenitor cells can promote cardiac repair and regeneration, but their mechanism has not been fully explained. Immune responses mediated by immune cells also play important and complicated roles in the progression of various cardiovascular diseases such as myocardial infarction and atherosclerosis. Exosomes derived from immune cells have shown pleiotropic effects on these pathological states, whether similar to or different from their parent cells. However, the underlying mechanism remains obscure. In this review, we first describe the biological characteristics and biogenesis of exosomes. Then we critically examine the emerging roles of exosomes in cardiovascular disease; the exosomes we focus on are derived from immune cells such as dendritic cells, macrophages, B cells, T cells, as well as neutrophils and mast cells. Among the cardiovascular diseases we discuss, we mainly focus on myocardial infarction and atherosclerosis. As active intercellular communicators, exosomes from immune cells may offer prospective diagnostic and therapeutic value in cardiovascular disease.

## Introduction

Although there has been a decline in cardiovascular disease (CVD) mortality over the past decade in a number of developed countries, CVD remains the leading cause of death in most middle-income countries (1) and some high-income countries. Globally, ischemic heart disease has become the leading contributor to the burden of disease as assessed by disability-adjusted life-years (2). Despite well-established initial treatments that mitigate acute cardiac damage of myocardial infarction via the restoration of normal anatomy, there is a need for novel therapies (3) to minimize subsequent cardiac remodeling and prevent the chronic progression of atherosclerosis. In addition, factors involved in the pathological response are being actively sought as novel means of diagnosis, especially in coronary artery diseases.

Exosomes are extracellular nanosized vesicles of endocytic origin that are actively secreted by most cell types. They are bound by a lipid bilayer of plasma membrane origin and contain multifarious cargos such as proteins, mRNAs, and miRNAs, shielding them from enzymatic degradation (4). Based on the constituents of exosomes, they are believed to transport intercellular information between tissue microenvironments (5). This has caused immense interest in their potential to act as therapeutic agents or biomarkers of diverse pathological states, especially CVD (4). Currently, the potential role of exosomes in cardiovascular diseases is being explored (6).

In terms of the cells that exosomes are derived from, in the context of CVD, growing attention is being focused on stem cells mobilized from bone marrow such as mesenchymal stem cells (MSCs) (7), hematopoietic stem cells (HSCs) (8), and cardiac progenitor cells (CPCs) (9). Exosomes derived from injured myocardial tissue or border-zone cardiomyocytes, and circulating plasma or serum are also frequently discussed (10). It is widely recognized that these exosomes are closely associated with cell-to-cell communication in the regeneration or repair process of the heart (8, 9). However, promising as they may be, their roles are complicated.

Taking myocardial infarction (MI) for example, the intense but transient inflammatory response triggered by MI is essential for cardiac repair, which clears the infarcted area of dead cells and extracellular matrix debris (11). There exists a sophisticated regulation of the balance between inflammatory reactions and the repression of inflammation, which prepares the infarcted area for the subsequent proliferative phase of healing. Immune cells, rather than injured cardiomyocytes, are the predominant modulators of this balance. A pivotal role is played in cardiac injury by cells that mediate innate immunity, such as neutrophils, mast cells, NK cells, monocytes, and more importantly, macrophages. Additionally, as professional antigen-presenting cells, macrophages are also involved in adaptive immunity, which involves dendritic cells, B cells, and T cells. Increasing evidence indicates that exosomes are released by these cell types both within the adaptive and innate immune systems (12). In spite of the paramount importance of exosomes from immune cells, present exosomal studies in CVD are in the minority.

Here, we critically review the emerging role of exosomes in CVDs. The exosomes we focus on are derived from classic immune cells such as dendritic cells, macrophages, B cells, T cells, as well as some non-professional immune cells like neutrophils and mast cells. We mainly discuss cardiovascular diseases such as atherosclerosis, cardiomyopathy, hypertensive endothelial dysfunction, and most interestingly, myocardial infarction. This knowledge could help in the development of prospective diagnostic markers and therapeutic approaches, which may benefit patients with CVDs.

## Biology of Exosomes

### Biogenesis of Exosomes and Related Vesicles

**Figure 1:**
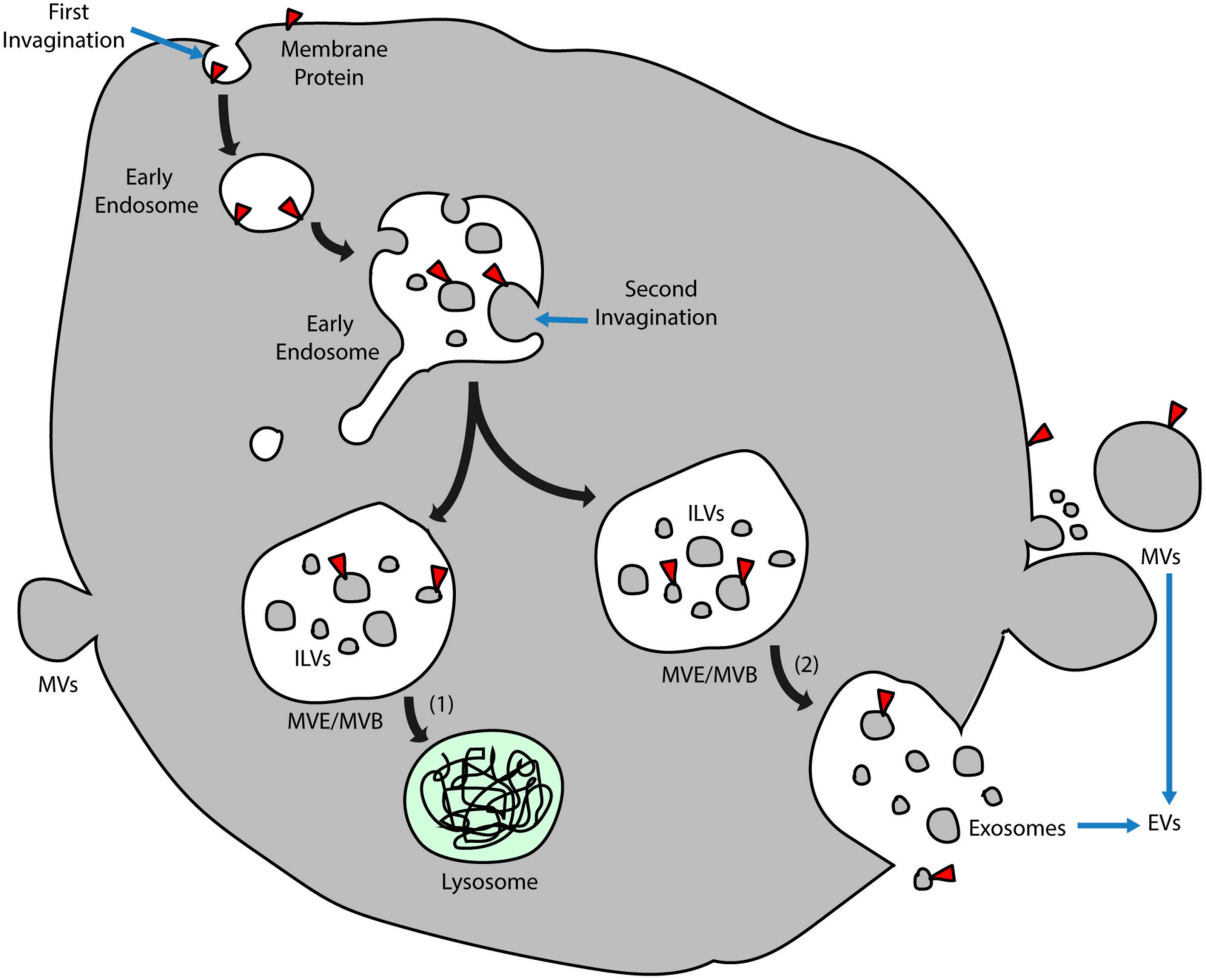
Exosome biogenesis. Early endosomes originate by inward budding of plasma membranes, and the orientation of membrane proteins is turned to the inside of the lumen. After a second invagination, small vesicles form inside the early endosomes during which the direction of membrane proteins turns again and coincides with that of the plasma membrane. These vesicles inside the lumen are known as ILVs, and endosomes that enclose the ILVs are called MVEs/MVBs. Two alternative MVE/MVB pathways include (1) ILVs transported for lysosome degradation and (2) MVE/MVBs coalescing with the plasma membrane and releasing ILVs into the extracellular fluid, which is named exosomes. Another group of EVs directly buds from the plasma membrane, which is called MVs. ILVs, intraluminal vesicles; MVs, microvesicles; MVE/MVB, multivesicular endosome/body; and EVs, extracellular vesicles.

Exosomes are nanometer-sized membrane vesicles released into extracellular fluids by cells. These vesicles, once overlooked as cell debris with no function, contain a rich source of cytosolic proteins, lipids, and nucleic acids, especially miRNAs, which are potentially functional and need to be fully explored. In retrospect, exosomes have been categorized as extracellular vesicles (EVs) that are subdivided into different categories depending on physical size, sub-cellular origin, morphology, and method of vesicle collection (10, 13). Despite various classifications, there are two main types of vesicle according to different mechanisms of EV formation (Figure 1). Those formed and released by direct budding from the plasma membrane are called microvesicles (MVs), which display a diverse range of sizes (100 nm to 1 μm in diameter), while those called exosomes are generated in more complicated multivesicular endosomes (MVEs) or multivesicular bodies (MVBs) and are secreted when the compartments fuse with the plasma membrane (14). The precise size of exosomes is controversial, from 30 to 100 nm (10) or roughly smaller than 150 nm (14), but they are more homogenous in size compared with MVs.

In addition to exosomes that originate from intraluminal vesicles (ILVs) contained in MVEs, another group of ILVs is delivered along the degradative pathway to lysosomes for further digestion (Figure 1). It has been reported that cone-shaped lipids inducing the formation of MVEs may differ in these vesicles of divergent destination, with lysobisphosphatidic acid determining the lysosome pathway and ceramide resulting in the exosome pathway (15). Nonetheless, it is still unclear how extracellular vesicles are generated under physiological conditions, whether in the form of exosomes or microvesicles or both. Besides, circulating exosomes are a mixture of those derived from various kinds of parental cells that are located in different organs and under different health conditions. Thus, differences between the biogenesis of exosomes under physiological and pathological states remain to be investigated.

### Constituents of Exosomes

Derived from early endosomes, exosomes possess surface proteins that partly originate from plasma membranes during endocytosis. Due to two successive invaginations from the plasma membrane (Figure 1), exosomes have the same membrane orientation as the plasma membrane (10); thus, the orientation of surface proteins on exosomes is the same as those on the plasma membrane. Although exosomes from many cell types may be characterized with similar surface proteins such as tetraspanins, membrane transport and fusion proteins, heat shock proteins, and proteins involved in MVEs biogenesis (10), currently there is a lack of widely accepted specific markers to distinguish exosomes from different populations of cells. Theoretically, functional membrane proteins should remain anchored on the surface of exosomes, such as cluster of differentiation (CD) molecules and major histocompatibility complex (MHC) molecules. In addition to membrane proteins transmitted via membrane invagination, some proteins are wrapped in during the formation of exosomes. In addition, the protein content is influenced by the cell source and stimulus for exosome formation (16). It is understood that exosomal protein composition is different from that of cells. In contrast, the RNA composition of exosomes might be similar to the parent cells or might be distinct depending on the cell types (17) and mainly comprises functional mRNAs as well as other non-coding RNAs, such as miRNAs and long non-coding RNAs (lncRNAs). Notably, while exosomal miRNAs have been extensively studied, lncRNAs from exosomes are only partly understood; they have a more complex structure and may transmit phenotypical functions such as drug resistance, angiogenesis, and tumorigenesis (18). Being able to interact with other coding and non-coding RNAs as well as proteins and DNA, the regulation of exosomal lncRNAs may be crucial in various diseases. How cytosolic cargos are recruited and segregated into the lumen of the early endosome not only depends on the endosomal sorting complex required for transport (ESCRT), which contains Hrs, Tsg101, Alix, and Vsg4, but also depends on raft-based microdomains containing ceramide, a sphingolipid generated by sphingomyelinase (15).

### Biological Properties of Exosomes

As vectors for intercellular communication, exosomes from donor cells induce subsequent physiological changes in recipient cells through binding to specific receptors, fusion of membranes, and release of their cargos. Some of the important functions of exosomes include promoting angiogenesis, tumor metastasis and disseminating malignancy (10), alleviating ischemia-reperfusion injury (19) and acting as antigen-presenting vesicles involved in stimulating T/B cells to induce either cellular adaptive immunity or humoral immunity (12). Furthermore, it has been proposed that several biological features of exosomes warrant attention, such as the unique protein/miRNA composition, the rigid lipid membrane protecting bioactive contents (particularly miRNAs) from degradation under adverse environments, the specific targeting and homing nature (20, 21), and the potential for manipulated drug delivery (22), especially in cardiac therapies.

### Isolation and Characterization of Exosomes

The physical features of exosomes are the basis of different isolation techniques (see Table 1). The extremely small diameter of exosomes makes it possible to separate by differential ultracentrifugation (final at 100 000 g) which gradually eliminates dead cells and cell debris and retains final pellet as relatively pure exosomes (23). That exosomes are capable of floating in density gradient supports another isolation method of ultracentrifugation in a sucrose gradient. Despite the high purity of exosomes and recognition as the gold standard isolation technique, the ultracentrifugation method is time-consuming and at high cost of special equipment, requiring large amounts of workloads and preparations, and the yields are limited to small volumes of samples (24, 25). In contrast, exosome precipitation technique utilizing water-excluding polymers to change the solubility of exosomes and facilitate them to precipitate is of high extraction efficiency and of ability to yield exosomes even from limited volumes of samples, which is widely used in commercial isolation kits for exosomes, such as Exoquick, Invitrogen Total Exosome Isolation Reagent (25, 26). The inevitable drawback of precipitation method is contaminations of other molecules that co-precipitate with exosomes, leaving the purity of exosomes questionable (24). When the purpose of isolating exosomes is for research use, the precipitation technique is cost-effective and guarantees the sufficient yields of exosomes for further extraction for RNA or analysis (26). Nevertheless, when it comes to clinical use for diagnosis or treatment, neither precipitation limited to purity issues nor ultracentrifugation constrained by yields is qualified for large-scale good manufacture practice. Ultrafiltration is another time-saving technique for isolating exosomes with filters of different size and molecular weight limits, but the integrity of exosomes is easier to be impaired (27), compared with size exclusion chromatography (SEC) which is also based on size exclusion (24). Chromatography will be a potential technique for clinical application only if the long run time issue is well-resolved (28). In addition, microfluidics-based devices have been fabricated for rapid and efficient isolation of exosomes, which is based on both physical and biochemical properties of these vesicles. As a new method, microfluidics technique is of potential for portable and fast test of exosomes, but the issue of standardization and validation remains to be accomplished (24). Selective isolation of exosomes offers a possibility that the precise regulation and interaction between highly purified and specific exosomes and target cells can be studied. However, none of the isolation methods above meets the requirements, except for immunoaffinity capture-based techniques. Based on binding of ligand and receptor, this approach can separate subpopulations from a mixture of exosomes from different origins. It is noteworthy that as the purity increases, the cost rises and yields decline in this isolating strategy. Besides, there is no widely recognized or gold-standard specific markers of exosomes for isolation at present, which hampers its application (28).

**Table 1:** Relative comparison of different techniques for isolation of exosomes.

**Technique**	**Run time**	**Work-load**	**General cost**	**Yields in small sample**	**Purity of exosomes**	**Specificity of exosomes**	**Integrity of exosomes**
Differential ultracentrifugation	Long	High	High	Limited	High	Low	Moderate
Density gradient ultracentrifugation	Long	High	High	Limited	High	Low	Moderate
Precipitation	Short	Low	Low	High	Low	Low	Moderate
Ultrafiltration	Short	Low	Low	High	Moderate	Low	Low
Size exclusion chromatography	Long	High	Moderate	Moderate	High	Low	High
Microfluidics	Short	Low	Low	Low	Low	Low	Moderate
Immunoaffinity capture	Long	High	High	Low	High	High	Moderate

In regard to characterization, initial electron microscopy analysis is typical in determining the size, with other approaches involving nanoparticle tracking analysis (NTA) and dynamic light scattering (DLS) analysis (10) based on the light scatter feature of exosomes. Although the light scatter is correlated to vesicles composition, flow cytometry (FC) analysis by now has not been taken into account because exosomes are too small to detect (13).

## The Potential of Exosomes as Biomarkers in Cardiovascular Diseases

### The Potential of Exosomal Biomarkers

A large number of studies have suggested that exosomes might be capable of reflecting the disease state according to their contents (29). It has been demonstrated that plasma exosome count, exosomal coding and non-coding RNAs, and exosomal proteins may serve as biomarkers for diagnosis and prognostic monitoring in cardiovascular diseases (see Table 2). For instance, the number of endothelium-derived microvesicles helps discriminate between patients with stable angina, first-time myocardial infarction, and recurring infarction (30). Moreover, exosomes containing miR-208a correlate well with cTnI levels after coronary artery bypass grafting (CABG) surgery, while miR-208a levels in blood circulation do not (31), indicating that exosomal miRNAs may be beneficial for diagnosing coronary artery diseases. In contrast, the protein contents of exosomes have had few studies for their diagnostic value, though they have been found to be reliable prognostic tools for predicting adverse cardiovascular events; examples include CD31+/Annexin V+ (32) and CD144-positive (33) extracellular vesicles. Exosomal miR-192, miR-194, and miR-34a have also been identified as prognostic biomarkers (34). Of note, miR-34a is highly expressed in the infarcted heart and is preferentially incorporated into exosomes released by cardiomyocytes and fibroblasts (35).

**Table 2:** Summary of exosomes as potential biomarkers in cardiovascular diseases.

**Exosome source**	**Markers**	**Disease/state**	**Diagnostic/prognostic**
Endothelium	Number	Stable angina, first-time myocardial infarction, recurring infarction	Diagnostic (30)
Plasma	miR-208a	Coronary artery bypass grafting	Prognostic (31)
Plasma	CD31+/Annexin V+	Stable coronary artery disease	Prognostic (32)
Endothelium	CD144+	Stable patients at high risk for coronary heart disease	Prognostic (33)
Serum	miR-192, miR-194, miR-34a	Heart failure after acute myocardial infarction	Prognostic (34)

### Pros and Cons of Exosomal Biomarkers Over Conventional Biomarkers

Testing exosomal biomarkers has been described as a form of “liquid biopsy” (36) that is less invasive and risky. Exosomes are similar to their cells of origin in some common markers, while they differ from parental cells and constantly alter the proportion of certain contents under different pathophysiological situations, which increases the accuracy of diagnosis at the molecular and cellular level. Thus, exosomes derived from immune cells can be considered convenient carriers that contain constitutively expressed immune system-specific proteins and nucleic acids that can be employed for the detection of immunologic status in cardiovascular diseases. Exosomes as well as their cargo including miRNAs that are usually prone to rapid degradation by RNAses (37) are stable over a period of time, allowing for the isolation and analysis of these miRNAs for diagnostic/prognostic purposes. The stability of exosomes and the protection of the cargo from degradation allows to isolate and analyze exosomes from multiple sources including blood, pericardial fluid, lymphatic fluid, and urine (36).

However, there are several technical limitations for clinical translation of exosomal biomarkers at present. The primary factor that hinders the clinical use is the lack of standardized pre-analytical and isolation procedures (36). Various isolation methods for exosomes have been adopted for research, but there is no possible method for the clear classification of all subpopulations of exosomes, and none of them is officially recognized and suitable for convenient and quick clinical testing. Utilizing different approaches to isolate exosomes from different originating cells and sources of fluids, it is tough to set convincing reference ranges under various circumstances. In addition, confounding factors like disease specificity and the presence of comorbidities and medications may have an influence on the level of exosomal biomarkers (36). More importantly, it remains to be further validated whether exosomes possess diagnostic and prognostic value for a large number of patients (38), and whether exosomes can provide additional value over current biomarkers that are widely and clinically adopted.

## The Potential of Exosomes as Therapeutics in Cardiovascular Diseases

**Figure 2:**
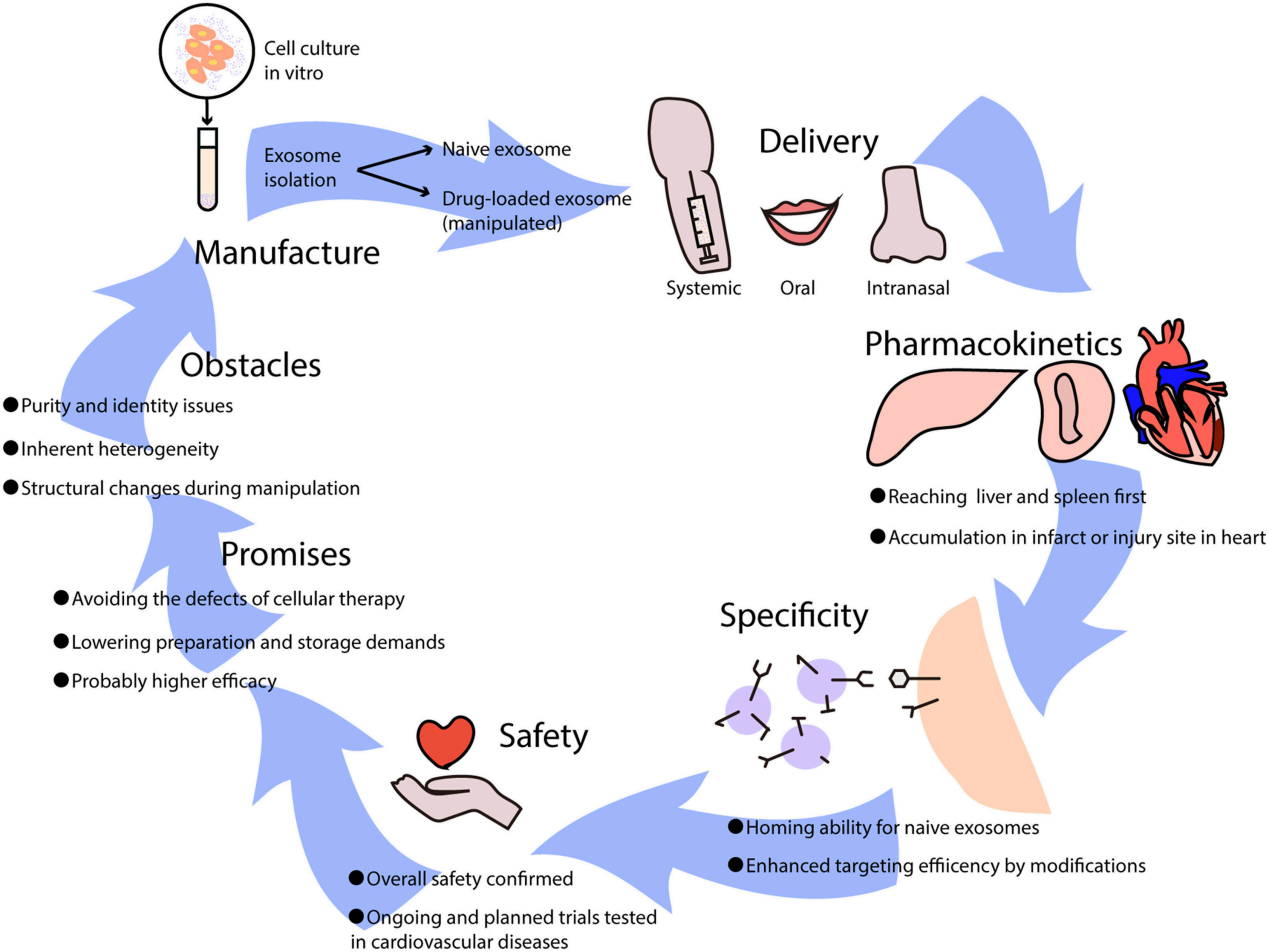
The potential use of exosome-based therapies in cardiovascular diseases. Exosomes used as therapeutics are usually isolated from cell culture *in vitro*, which can be divided into naïve exosomes and drug-loaded exosomes that undergo artificial manipulation. Exosomes can be delivered into the body via intravenous (systemic) administration, oral administration, and intranasal administration, leading to different biodistributions and effects. When exosomes come into circulation, they first reach the liver and spleen where monocytes are located, and they may be recruited to infarcts or injury sites in the heart. Some naïve exosomes possess homing abilities, while others lack a unique target and can be improved by adding modifications. It has been reported that exosome-based therapies are safe overall, though further clinical trials are needed to test cardiovascular diseases. Generally, the promises of exosome-based therapeutics are huge, avoiding the difficulties that cellular therapies have, lowering manufacture costs, and increasing efficacy in treating diseases. However, certain obstacles need to be resolved such as purity and identity issues, inherent heterogeneity, and structural changes during manipulation.

Preclinical studies have demonstrated the protective effects of exosomes in ischemic heart diseases via alleviating myocardial ischemia-reperfusion injury, and by promoting angiogenesis and cardiac regeneration (39). Generally, exosome-based therapies can be divided into two categories according to whether modifications or drugs are added (Figure 2). Naïve exosomes released directly from parental cells may exert protective and regenerative effects on recipient cells, and those derived from immune cells are more likely to possess immune-modulating abilities (40), which implies their therapeutic potential for moderating immune responses elicited in cardiovascular diseases. However, it is difficult to manipulate naïve exosomes because of their multiple biological effects, thus an increasing number of researchers have been attempting to rebuild exosomes by incorporating low-molecular-weight drugs or by modifying their parental cells (40). Exosomes can be loaded with drugs by incubation at room temperature, electroporation, and mild sonication (41), which surprisingly enhances the efficacy and release efficiency of drugs. Furthermore, pretreatment of parental cells with therapeutic agents and genetic modification of donor cells may help in targeting exosomes to lesions and have been shown to efficiently deliver exosomes with specific modifications (41).

### Delivery and Pharmacokinetics

In preclinical studies of the pharmacodynamics of exosomes, it has been recognized that their biodistribution varies according to source cell type and delivery approach, with predominant distribution in the liver. MSC-derived exosomes can reach the brain, liver, lung, and spleen after intravenous injection (42), while exosomes derived from mature dendritic cells (DCs) mainly accumulate in the liver and spleen and peak at 4 h after injection (21, 43); this resembles the innate homing ability of mature DCs that migrate to peripheral lymphoid organs. With a similar half-life ranging from minutes to hours due to rapid sequestration by the mononuclear phagocytic system (MPS) (44), systemically applied exosomes also resemble their parental cells in their pharmacokinetics (45). Nevertheless, intranasal delivery has resulted in better brain accumulation of exosomes compared to intravenous administration (46). Aside from delivery approaches that influence the distribution, exosomes can also be recruited to injury sites, such as myocardial infarcts (43) or livers undergoing ischemia-reperfusion injury (19). However, the primary biodistribution of exosomes in the liver and kidney upon administration has led researchers to pursue reliable delivery or targeting strategies that can provide adequate concentrations in targeted areas. Of note, clinical trials that have focused on the biodistribution of exosomes are extraordinary scarce (47), and it remains to be studied whether clinical findings are in conformity with preclinical data.

### Specificity, Clinical Testing, and Safety

It is known that exosomes are excreted from original cells and accumulate in areas where target cells are located, which may not be limited to merely single cell type. As has been mentioned, exosomes of immune cells may possess homing abilities and accumulate in specific tissues such as the spleen and lymph nodes. Thus, the effects of naïve exosomes may be multidirectional, indicating that the specificity of naïve exosome-based therapies may be in doubt. However, other unknown targets of exosomes make no difference as long as they are efficacious toward the target cells that we focus on, and positive outcomes by using naïve exosomes have been proven in several clinical trials (48). Additionally, the specificity and targeting efficiency of exosome-mediated delivery can be improved in various ways, such as with the conjugation of antibodies or nanobodies, the enrichment of target-specific receptors or ligands, and magnetization of exosomes to help increase accumulation in specific tissues (49). Nevertheless, as the targeting mechanism is currently vague and unclear, targeting specific diseases calls for further investigation.

A small number of phase I clinical trials have revealed the overall safety (50) and considerable stability of exosomes derived from different cell types in circulation. So far, clinical trials using exosomes for treatment of cardiovascular disease have not started (51), and safety, as well as feasibility of exosome-based therapeutics for the heart, remains to be validated. It is known that exosomes are key mediators in the regenerative effects of cardiosphere-derived cells, and clinical testing has been planned (52). Meanwhile, several companies have established exosome products for cardiological diseases, awaiting support from future clinical trials, in which the exosomes are mostly derived from regenerative stem cells. DC-derived exosomes have drawn particular attention and have shown safety and efficacy in a number of phases I and II clinical trials (48, 53, 54), especially for malignancies such as melanoma and non-small cell lung cancer. The reason why DC-exosomes are of interest in cancer immunotherapies is that they can shape the quality of therapy-associated antitumor immunity when employed directly or they can indirectly exert anti-tumor effects by checkpoint blockade (55). It is the same situation in cardiovascular disease-evoked immune reactions that also rely on DCs; thus, exosomes from DCs and other immune cells may have potential in the treatment of cardiac injury and inflammation.

### Promises and Obstacles

Cell-free therapies based on exosomes that mimic and even exceed the effects of cellular therapies have various advantages. Above all, the problems of cell-based therapies are the potential for cellular embolism after intravenous administration and difficulty of reaching the target area through the blood-brain barrier, which can be avoided by exosomes (45). From the perspective of the manufacturing process, exosome-based therapies simplify preparation as well as sterilization and have fewer storage demands (50), substantially reducing the overall cost compared to stem cell therapies. Additionally, it is more flexible to utilize exosomes by targeted engineering or incorporating drugs compared to their cellular counterparts. Apart from their therapeutic value, exosomes are potential diagnostic or prognostic biomarkers. With exosomes being increasingly researched and applied to cardiovascular diseases such as myocardial infarction (50), it can be envisaged that based on well-established cellular immunotherapies, exosomes from immune cells will represent new efficacious alternatives for patients.

However, there are obvious shortcomings, primarily purity and identity issues due to existing limited separation methods. After exosomes or extracellular vesicles from tissues or expanded cell cultures are manufactured, it is inevitable that these products contain other kinds of soluble and unpackaged components, such as proteins, lipids, and nucleic acids, which hampers the testing of pure exosomes (50). In addition, clinical applications are hindered by the inherent heterogeneity of separated products that may contain microvesicles varying in a range of diameters and sizes. To resolve this conundrum, it has been proposed that the heterogeneity of secretome-based preparations should be accepted, and to redefine extracellular vesicles to get around the stringent demands for purity and identity, such as by using vesicular secretome fractions (VSFs) (50). The second challenge is whether the number of exosomes used for therapies is sufficient, because a different choice of the parental cell leads to different exosome yields. Another problem is the permeabilization procedure, which impairs the integrity and immune-privileged status of exosomes and makes drug-loaded exosomes easier to be degraded by macrophages (40). Moreover, exosome-based therapeutics can also be impacted by the parental cell type, modifications in handling, culture conditions, and materials or medical devices used for administration (50) which adds to the complexity of clinical usage. Therefore, future studies should solve the two major challenges: how to preserve structure and content during modification of exosomal carriers, and how to develop stable and efficient good manufacturing practice (GMP)-grade methods for large-scale manufacture.

## Dendritic Cell-derived Exosomes

Dendritic cells (DCs) are an essential member of the professional antigen-presenting cells and have the unique and strong ability to present peptides derived from exogenous proteins that are acquired by endocytosis (12). DCs have been reported to release EVs, which are seen as non-negligible intercellular communicators in adaptive immunity. Given the potential of DCs in modulating immune responses, a majority of studies of DC-derived exosomes have been focused on immunotherapy against cancer, which has translated into some clinical benefit (56–58). Nonetheless, exosomes from DCs also play important roles in cardiovascular diseases like acute myocardial infarction, autoimmune diseases such as myasthenia gravis (MG) (59), and transplantation research (60).

There are remarkable similarities between DCs and DC-produced exosomes in regard to membrane proteins and biological properties. CCR7, a chemokine receptor guiding mature DCs to peripheral lymphoid tissues like spleen, has been found to analogously regulate the increased accumulation of exosomes derived from mature DCs in spleen and inflammatory responses upon injection in mice (21). Like DCs, the molecular composition of their exosomes includes surface expression of functional MHC-peptide complexes, T cell costimulatory molecules (61) and other components that interact with immune cells. Antigen presentation by DC-derived exosomes can either be direct or dependent on bystander DCs whose plasma membrane remains associated with the released exosomes (12). Mounting evidence suggests that these exosomes have the potential to facilitate immune cell-dependent tumor rejection (48); likewise, they have advantages over cardioprotective immunotherapies involving DCs.

### Myocardial Infarction

As hypoxia and reoxygenation can simulate ischemia-reperfusion (62), hypoxic or necrotic cardiomyocytes simulate the post-MI microenvironment to release exosomes from bone marrow-derived DCs (BMDCs) in mice, and cardiac function is improved after the injection of exosomes under left coronary ligation (43). In addition, exosomes from MI-treated DCs are largely recruited to the spleen and uptaken by CD4^+^ T cells, which are activated and have increased expression of cytokines (43). Given that the frequency of anti-inflammatory CD4^+^ Foxp3^+^ Tregs among all CD4^+^ T cells in heart-draining lymph nodes has been demonstrated to increase as early as 3 days after MI (63), it is speculated that DC-derived exosomes might activate cardioprotective Tregs after infarction. In addition, both ablation of regulatory dendritic cells and depletion of Tregs in diphtheria toxin receptor (DTR) transgenic mice showed deteriorated left ventricular function and remodeling after MI, with aggravated cardiac inflammation, which is likely to be responsible for adverse clinical outcomes (64, 65). In contrast, therapeutic Treg-cell activation after MI reversed the outcome and improved healing, emphasizing that Tregs may be the downstream regulatory target of DCs and DC-derived exosomes in MI. It has been reported that administration of infarct lysate-primed tolerogenic DCs induces infarct tissue-specific Treg populations systemically and improves postinfarct left ventricular remodeling in MI mice (66), confirming the relationship between DCs and Tregs and the feasibility of using DC-derived exosomes to induce Tregs in post-MI remodeling. Generally, infarct lysate-pulsed DC-derived exosomes display therapeutic effects of remodeling after MI, in which the mechanism may involve activation and proliferation of CD4^+^ Foxp3^+^ Tregs.

### Ischemia-Reperfusion Injury (IRI)

There have been few studies on DC-derived exosomes involving myocardial ischemia-reperfusion injury (IRI), but they have demonstrated that exosomes produced by BMDC can significantly alleviate hepatic IRI via modulating the balance between Tregs and Th17 cells, which results from heat shock protein 70 kDa (HSP70) transported by exosomes that stimulate the PI3K/mTOR axis (19). Considering the powerful cardioprotective effects of plasma exosomes in models of cardiac IRI, and due to HSP70 that was enriched in these circulating exosomes (67), it is highly probable that DC-derived exosomes display similar phenotypes and potentially account for a considerable fraction of total exosomes in blood.

### Atherosclerosis

Exosomes derived from mature DCs stimulated by other antigens seem to be atherogenic. After a period of 12 weeks of lipopolysaccharide-treated mature DC-exosomes injection into ApoE^−/−^ mice, atherosclerotic lesions significantly increased, with endothelial cell inflammation activated via the membrane tumor necrosis factor-α (TNF-α)-mediated NF-κB pathway (68, 69). However, exosomes from distinct DC subsets might contribute to different outcomes. It has been reported that common DC precursors differentiate into classical DCs (cDCs) and plasmacytoid DCs (pDCs), and cDCs can be further segregated into cDC type 1 (cDC1s), which encompass CD8α^+^ DCs and CD103^+^ DCs, and CD11b^+^ cDC type 2 (cDC2s). Among all these subsets, some exert protective functions in atherosclerosis development, for instance, CD103^+^ classical DCs; other subsets such as chemokine ligand CC family (CCL17)-expressing CD11b^+^ DCs restrain Treg responses and drive atherosclerosis. Additionally, certain signaling machinery in DCs is critical to the suppression of atherosclerosis, such as transforming growth factor-β (TGF-β) type II receptor and toll-like receptor (TLR) adaptor Myd88 (70). Most of these protective effects appear to be closely related to promoting Treg activity and dampening proinflammatory effector T cell activity. Therefore, the atherogenic feature of mature DC exosomes could be attributed to the subset of DCs that mainly promote inflammation and endothelial dysfunction. It is of interest whether exosomes from DCs that undergo stimulation by other unknown antigens have reversed functions.

### Macrophage-Derived Exosomes

Macrophages are derived from the monocyte cell lineage, which is heterogeneous (71), and healing and remodeling after cardiac injury, especially myocardial infarction, involve the successive recruitment of different macrophage subsets with complementary functions. During the first few days after MI, the number of M1 macrophages peaks, and these cells are considered to be highly phagocytic and pro-inflammatory (72). Afterward, macrophages in the infarct shift from M1 to M2, which is believed to tune the anti-inflammatory and reparative response (71). Macrophages can also release abundant amounts of exosomes, and these exosomes have a tendency to be pro-inflammatory in various contexts, partly due to previously primed or activated macrophages that have already been primed with stimuli. So far there have been only a few studies (73) rigorously distinguishing between exosomes from distinct types of macrophages (mainly M1 and M2). The immunologic function of exosomes from macrophages acting as vehicles carrying antigen-presenting molecules has been proposed (74, 75), and may rely on the presence of DCs (76).

## Myocardial Infarction

Unlike DCs, macrophage-derived exosomes containing miR-155 are adverse to cardiac repair after MI. It has been suggested that exosomes from macrophages transfer miR-155 into cardiac fibroblasts, inhibiting fibroblast proliferation and enhancing inflammation. The effects mediated by miR-155 have been elucidated with *in vivo* experiments creating miR-155-deficient mice, which display a significant reduction in the incidence of cardiac rupture and improved cardiac function (77). In addition, it is noteworthy that macrophages themselves are also recipients of miR-155-enriched exosomes from endothelial cells, which further alters macrophage polarization (78). Other than exosomal miR-155, several pro-inflammatory miRNAs (miR-19, miR-21, miR-146, and miR-223) are also increased in the total microparticles from patients with acute coronary syndrome (ACS), as contrasted with patients with stable coronary artery disease (CAD) (79), which may provide insights for detecting other miRNAs in macrophage-derived exosomes.

### Sepsis-Induced Cardiomyopathy

Macrophage-derived exosomes typically enhance inflammation and damage to the heart. In sepsis-induced cardiac inflammation, the release from exosomes of pro-inflammatory cytokines is impaired in RAW264.7 macrophages pre-treated with GW4869, which is an inhibitor of sphingomyelinase and capable of inhibiting exosome biogenesis/release. Accordingly, GW4869 treatment diminishes the sepsis-triggered inflammatory response, attenuates cardiac dysfunction, and prolongs survival in mice (80), emphasizing the critical role of exosomes in an inflamed heart.

### Atherosclerosis

Pro-inflammatory macrophages can indirectly exacerbate both atherosclerotic and aortic valve calcification via the secretion of inflammatory cytokines such as IL-6 and TNF-α (81). Elastolytic cathepsins and matrix metalloproteinase (MMPs) that promote collagen degradation are also released by macrophages, which further cause extracellular matrix (ECM) degradation and remodeling, leading to lethal plaque instability and rupture (82). Given that macrophages contribute to both vascular and valvular calcification at the same time, it is speculated that exosomes secreted by macrophages might act as a dynamic calcifying factor. According to Bakhshian et al. (83), the accumulation and aggregation of macrophage-derived EVs result in mineral growth within atherosclerotic plaques, confirming this assumption. Of note, whether exosomes can also give rise to lesion ruptures or instability of macrophages has not been clarified. When macrophages transform into foam cells in lesions, foam cell-derived EVs promote increased levels of vascular smooth muscle cell (SMC) migration and adhesion, and regulate the cytoskeleton and focal adhesion pathways to a greater extent than macrophage-derived EVs (84), which may be mediated by the integration of EVs into vascular SMCs and the subsequent downstream activation of ERK and Akt (85). This may be one mechanism by which exosomes accelerate the development of atherosclerotic plaques.

### Hypertension and Endothelial Dysfunction

It has been suggested that macrophage-derived exosomes, at least partly, can elicit inflammation of endothelial cells (ECs) under hypertensive conditions. Using a macrophage cell line, human THP-1, Osada-Oka et al. (86) isolated the exosomes both from angiotensin II (Ang II)-infused rat serum and Ang II-stimulated THP-1 cells and compared the effects on the expression of inflammatory factors in ECs. Both *in vivo* and *in vitro* tests showed enhanced expression of intracellular adhesion molecule-1 (ICAM-1) and plasminogen activator inhibitor type 1 (PAI-1) in ECs, with decreased miR-17, an inhibitor of ICAM-1 expression. Likewise, monocytes induced with different antigens showed distinct exosome altering of adhesion molecule expression in ECs. It has been demonstrated that both LPS and LPS+IFN-α stimulated monocyte-derived exosomes are able to significantly increase ICAM-1, CCL-2, and IL-6 expression, deteriorating EC dysfunction via TLR4 and NF-κB pathways, compared with exosomes from cells treated with IFN-α alone or unstimulated monocytes (87). Unesterified cholesterol (UC) triggers procoagulant MVs from monocyte/macrophages with analogously increased ICAM-1 and augments the adhesion of monocytes to ECs (88). Such results are similar to DC-derived exosomes that perform disparate roles depending on the antigens that stimulate the cell of origin. Collectively, the exosomes from macrophages might contribute to EC dysfunction and injury, posing potential risks for target organ damage in the context of hypertension.

However, it is interesting that THP-1-derived exosomes have been shown to increase human HMEC-1 EC migration in transwell assays, with the potential transfer of miR-150 from exosomes secreted by macrophages to ECs (89). These findings make it conceivable that macrophages under some circumstances could protect and influence EC migration and angiogenesis through exosome secretion (90).

## B Cell-derived Exosomes

Exosomes from both human and murine B cell lines carry MHC-II-peptide (pMHC-II) complexes, as well as costimulatory and adhesion molecules, demonstrating the ability to induce pMHC-II-restricted T cell responses and antigen-presenting capacities just like B cells (91). Different types of antigens, carried by exosomes from B cells, may dictate different types of immune responses (5). One of the potential targets of B cell-derived exosomes is follicular DCs, which are abundantly decorated at their plasma membranes with MHC-II-carrying exosomes in lymphoid follicles (12). Other evidence supports the idea that B cell-derived exosomes traffic to the subscapsular sinuses of lymph nodes and associate with CD169^+^ macrophages, which further limits the dissemination of viruses or tumor cells (92). In respect to the CD8 T cell-mediated response to B cell-derived exosomes, B cell depletion or lack of secreted and membrane-bound antibody (Ab) does not alter the exosome-induced cytotoxic T lymphocyte (CTL) response (93). In addition, CD4 T cells are essential drivers of B cell affinity maturation and the development of memory B cells (94), which involves exosome-mediated communication. Nevertheless, few preclinical studies concerning exosomes from B cells have focused on cardiovascular disease.

Considering the natural ability of some B lymphocytes to express the death-inducing molecule Fas ligand (FasL), it has been shown that Epstein-Barr virus (EBV)-immortalized human lymphoblastoid cell lines can be used as cellular factories for FasL (+) MHCII (+) exosomes, which have important roles in natural immune tolerance and can be employed to establish tolerance toward specific antigens (95). Of note, TLR3 deficiency shows significant attenuation of cardiac dysfunction after MI of IRI; meanwhile, levels of Fas, Fas ligand or CD95L (FasL), Fas-associated protein with death domain (FADD), Bax, and Bak in myocardium are attenuated compared to their increase after IRI (96). Tolerogenic exosomes derived from B cells may have a great deal of therapeutic potential via targeting Fas or other antigens involved in apoptosis of cardiomyocytes or T cells (97), which contributes to detrimental inflammation after cardiac injury.

## T Cell-derived Exosomes

T cells, like the professional antigen-presenting cells (APCs) they interact with, release exosomes with heterogeneous characteristics. These vesicles can be targeted to different sorts of immune cells and modify their function (12). Th1-cell delivery of help to B cells is based on contact-dependent CD40L transfer (98), which implies a role for T cell-derived exosomes during the transfer process. Typically, in relation to DC-T cell interaction, T cell exosomes are released upon T cell activation by DCs. Specifically, T cells could prime DCs via the transfer of exosomal DNA, making DCs more protective against pathogen infection (99). It is highlighted that DNAs in exosomes derived from T cells, other than non-coding RNAs, may confer significant signals to immune system when encountering threats. A specific group of activated T cell-derived exosomes can modulate the activity of immune cells, including other T cells (100, 101). In addition, the activation status of CD4^+^ T cells regulates the release of distinct vesicle subpopulations with various abilities to activate other untouched T cells (102). The protein expression profile of T cell exosomes can also be changed substantially after the induction of apoptosis. Apoptotic T cells release more apoptotic bodies than exosomes, while activated T cells release exosomes and MVs both in lower amounts (103).

Similar to B lymphocyte-derived exosomes, in T lymphocytes exosomes containing FasL are also secreted, known as “lethal exosomes,” following activation-induced fusion of the MVB with the plasma membrane (104). Analogously, it has been proposed that these exosomes help prevent potential autoimmune damage by eliminating activated T cells (105, 106). This indicates that it is likely that these exosomes carrying FasL have a protective impact on the clearance of dead debris of cardiomyocytes as well as restriction of injury-associated inflammation post-MI.

Activated T cell-derived exosomes have been demonstrated to correlate with an endothelial injury. Anti-CD3/CD28 Ab-activated T cells release exosomes containing miR-142-3p, which can be transferred to human vascular endothelial cells and result in increased endothelial permeability. Thus, the role of T cell-exosomes explains at least some of the reason for acute cellular rejection in heart transplantation, which manifests high miR-142-3p in exosomes isolated from patients' serum (107). Apart from miRNAs, thrombospondin-1 receptor CD47 has been found to be expressed on exosomes derived from T cells, regulating endothelial cell responses to vascular endothelial growth factor (VEGF) and tube formation (108). Moreover, MVs generated from T cells undergoing activation and apoptosis prevent actinomycin D-induced apoptosis of ECs by modulating reactive oxygen species (ROS) production (109).

## Exosomes From Myeloid Derived Suppressor Cells (MDSC)

Apart from regulatory T cells (Tregs), myeloid derived suppressor cells (MDSC) represent another subset as immune suppressor. MDSCs originate from immature myeloid cells under pathological microenvironment, comprising a heterogenous population of CD11b^+^Gr-1^+^ cells including myeloid cell progenitors and precursors of granulocytes, macrophages and dendritic cells (110). Generally, MDSCs possess strong immunosuppressive ability against effector cells, mainly effector T cells and other pro-inflammatory cells. Whereas, two subpopulations of MDSCs, CD11b^+^LY6G^−^LY6C^high^ monocytic-MDSCs (M-MDSCs) and CD11b^+^LY6G^+^LY6C^low^ granulocytic-MDSCs (G-MDSCs), vary in their suppressive capability. It has been reported that M-MDSCs could exert stronger inhibition against T cell activation than G-MDSCs both *in vivo* and *in vitro* (111, 112). Intriguingly, the inflammatory milieu and exogenous stimuli that facilitate the differentiation from immature myeloid cells to MDSCs can stimulate the release of exosomes from MDSCs (113), which carry various cargos of known or predicted functions in accordance with the immunosuppressive activity of their parental cells (114). The suppressive functions of MDSC-derived exosomes against T cell proliferation and promoting Tregs expansion have been validated in dextran sulfate sodium (DSS)-induced colitis in mice (115). Besides, mammary carcinoma-primed MDSCs could release exosomes that implement some of the tumor-promoting functions of MDSCs (116). But whether exosomes from MDSCs and their subsets have direct influences on cardiovascular diseases remains unclear.

It has now become increasingly clear that MDSCs develop along with atherosclerosis and display suppressive effects of pro-inflammatory responses with reduced atherosclerotic plaques, as validated in both LDLr^−/−^ and ApoE^−/−^ mice models for atherosclerosis (112, 117). Specifically, oral administration of HSP60 resulted in reduction of plaques and increase of M-MDSCs in ApoE deficient mice, while subcutaneous immunization of HSP60 caused contrary effects and increase of G-MDSCs (112). It is evident that the stronger suppressive ability of M-MDSCs than G-MDSCs may account for the different outcomes. Thus, it is possible that exosomes from M-MDSCs may differ from those released by G-MDSCs in their functions and influences on atherosclerosis, but it remains to be elucidated by further studies. In addition, it has been demonstrated that doxorubicin-treated breast tumor bearing mice could induce the differentiation of MDSCs, which release exosomes containing miR-126a and lead to tumor angiogenesis and metastasis (113). On the other hand, administration of miR-126-5p could rescue endothelial function and proliferation and therefore limit the atherosclerotic lesion formation (118), indicating that MDSC-derived exosomal miR-126a might possess the same anti-atherosclerosis ability.

## Neutrophil- & Mast Cell-derived Exosomes

In the early hours after myocardial infarction, leukocytes comprising neutrophils and mononuclear cells rapidly infiltrate the infarct (11). Additionally, cardiac resident mast cells containing preformed proinflammatory cytokines can rapidly release their granular content and trigger the inflammatory cascade (119). In the light of the proinflammatory characteristics of neutrophils and mast cells after acute myocardial injury, it has been speculated that neutrophil-derived exosomes and mast cell-derived exosomes play important roles in initiating innate immunity in response to damage-associated molecular patterns (DAMPs).

Neutrophil-derived exosomes can modulate the activities of smooth muscle cells (SMCs). Compared to unstimulated neutrophil exosomes, those exosomes from neutrophils after stimulation with LPS carry proteins associated with immune responses and positive regulation of cell communication, demonstrating altered proliferative properties of airway smooth muscle (ASM) cells. This implies that neutrophil-derived exosomes can play an important role in the progression of asthma and promoting airway remodeling (120), which provides inspiration for examining their roles in cardiac remodeling, especially after myocardial infarction.

Mast cell-derived exosomes have intimate relationships with multiple cell types, including T and B lymphocytes (121), dendritic cells (122) as well as other mast cells (123). Concerning T cells, mast cell-secreted exosomes derived from bone marrow partially promote the proliferation and dramatically enhance the differentiation of naïve CD4^+^ T cells to Th2 cells (124), presenting an immunomodulating orientation of the Th2 type. It is of note that mast cell-derived exosomes via significant upregulation of PAI-1 expression on endothelial cells may provide feedback between the characteristically increased PAI-1 levels and procoagulant states, both observed in diverse syndromes manifesting as endothelial dysfunction (125).

## Future Perspectives

### Implications for DC-Exosomes From Autoimmune Diseases and Cancer Research

In experimental autoimmune myasthenia gravis (MG) mice, exosomes from DCs overexpressing miRNA-146a suppress ongoing clinical MG and alter T helper cell profiles from Th1/Th17 to Th2/Treg (59). Another autoimmune disease, inflammatory bowel disease (IBD), which is caused mainly by excessive inflammation from exogenous antigens, has been shown to be improved after the injection of *S. japonicum* soluble egg antigen (SEA)-treated DC exosomes compared to untreated DC exosomes. The mechanism underlying this phenomenon might involve inducing tolerance to antigens, promoting epithelial barrier function (126), facilitating Treg expansion, and inhibiting Th1 cell proliferation (115), but it remains to be further clarified.

DCs pulsed with tumor antigens are known to facilitate antitumor immune responses, as do exosomes. Alpha-fetoprotein (AFP)-expressing DCs secrete exosomes that activate more interferon-γ (IFN-γ)-expressing CD8^+^ T cells while downregulating Tregs secreting interleukin-10 (IL-10) and TGF-β, mediating an antigen-specific immune response and presenting antitumor efficacy in hepatocellular carcinoma (127). Using autologous DC exosomes loaded with melanoma-associated antigens (MAGE), a phase I clinical trial has been completed in cancer patients that shows an increase in systemic responses against MAGE in some patients (128). Other ongoing clinical trials indicate that DC-derived exosomes can be developed for use as cell-free cancer vaccines (58).

Inspired by autoimmune disease and cancer therapies using DC exosomes, and to contextualize the functions of exosomes in cardiac pathophysiology, we assume that the antigenic components of DCs in contact with cells may have a decisive position. Antigens like exogenous lipopolysaccharide (LPS) might finally result in the expansion of atherosclerosis because of exacerbated inflammation. Alternatively, beneficial effects are witnessed when endogenous antigens are loaded into DCs, such as infarcted myocardium lysate, or with no antigen at all. It is unclear which antigen in the complex components of infarct lysate actually accounts for the outcome. Moreover, it is assumed that exosomes can be engineered to obtain the best clinical efficacy against cardiovascular diseases by manipulation of antigen-primed DCs, though further studies are required to clarify this.

It is also of note that the multifarious subsets of DCs have an influence on the functions of exosomes and their relationship with the activation of different subsets of T cells, requiring more research effort. Furthermore, immature or suppressive DC-derived exosomes harbor anti-inflammatory properties distinct from mature DC-derived exosomes, and can reduce T cell-dependent immunoactivation in murine models of autoimmune diseases and transplantation (129, 130). It has been reported that there is a discrepant composition of exosomal miRNAs: exosomes from immature DCs carry more miR-21, miR-34a, miR-221, and miR-222; exosomes from mature DCs contain more miR-125b-5p, miR-146a, miR-148, and miR-155 (131). Thus, exploring the roles of selected miRNAs in specific exosome subpopulations could lead to a comprehensive understanding of their clinical application and underlying mechanisms.

### Implications of Exosomes From Regulatory T Cells (Tregs)

As has been explained before, Tregs can improve healing and remodeling after myocardial infarction (63, 65, 66) and have other cardioprotective effects like delaying the progression of atherosclerosis (70). But it seems that Treg-derived exosomes are more recognized and prospectively applicable in organ transplantation than cardiovascular diseases (132–135).

DC exosomes having benefits for the heart might rely on Treg activation. However, if it is possible for exosomes from Tregs to be included in the downstream pathways involved in cardioprotection remains elusive. A unique population of CD4^+^CD25^−^ Tregs (dnlKK2-Treg) has shown inhibitive effects without needing cell-to-cell contact against the rejection of kidney allografts, in which exosomes from these specific Tregs account for the same immunosuppressive activity as their parent cells (134). Exosomes derived from another group of CD4^+^CD25^+^Tregs exhibit similar postponed allograft rejection and prolonged survival time of transplanted kidneys, as well as suppress T cell proliferation (135). Although specific subsets of effector Tregs that restrict cardiac inflammation may be unrecognized, they may share the cell-free modality mediated by exosomes.

However, the mechanistic basis of suppression via Treg-derived exosomes is not understood completely. It has been demonstrated that Tregs suppress other T cells such as Th1 cells, which rely on transferring exosomal miRNAs to recipient cells (136). On the other side, CD73 present on Treg-derived exosomes is essential for their suppressive function (137). In T CD8(+) suppressor (Ts) cells, however, the generation and action of Ts cell regulatory exosomes have been shown to be substantially influenced by macrophages (138). In turn, when treated with Ts cell-derived exosomes, macrophages can enhance the production of reactive oxygen intermediates (ROIs) (139). It is notable that activated ovalbumin (OVA)-specific CD4^+^ T cells produce exosomes that inhibit DC-stimulated *in vitro* CD4^+^ T cell proliferation and *in vivo* CD8^+^ CTL responses to tumor cells (140). Therefore, it is possible that the antigen-priming precursors of Tregs account for the immunosuppressive property of exosomes derived from them.

## Conclusion

Immune cells, unlike cardiomyocytes and stem/progenitor cells, are of paramount importance to cardiac immune responses and increased inflammation in various cardiovascular diseases. The discovery of the functions of exosomes secreted by these immune cells has complicated research on the role of different immune cells in the progression of heart disease (Table 3). On top of that, only a small fraction of exosomes in immune regulation have been discovered (12), and it is difficult to infer the possible applications of these exosomes in cardiac repair due to a lack of studies in this area. Nevertheless, several studies have demonstrated cardioprotective effects of exosomes from immune cells. In addition, overwhelming and increasing evidence for the prominent functions of exosomes implies future progress.

**Table 3:** Summary of exosomes derived from immune cells in cardiovascular diseases.

**Source**		**Target cell**	**Effects**	**Mechanisms**	**References**
DCs	BMDCs (stimulated by necrotic cardiomyocytes)	CD4^+^ T cells	Post-MI cardiac function and left ventricular remodeling are improved	It is possible that Tregs (66) activated could reduce cardiac inflammation	(43)
	BMDCs (stimulated by LPS)	ECs	Atherosclerotic lesions are significantly increased	Membrane TNF-α on the surface of exosomes mediated NF-κB pathway	(68)
Macrophages	Bone marrow-derived macrophages (stimulated by Ang II)	Cardiac fibroblasts	The incidence of cardiac rupture post-MI is increased	Exosomal miR-155 was transferred and inhibited fibroblast proliferation and enhanced inflammation	(77)
	RAW264.7 macrophages (stimulated by LPS)	Naïve macrophages	Sepsis-induced inflammatory responses and cardiac dysfunction can be reversed after the inhibition of exosome release	Exosomes possessing higher quantities of pro-inflammatory cytokines may act as signaling molecules	(80)
	^*^RAW264.7 macrophages (stimulated by Ca/P)	Unknown	Accumulation and aggregation of exosomes and MVs result in mineral growth within atherosclerotic plaques.	S100A9 and annexin V on the surface of MVs form a complex and accelerate the nucleation of MVs.	(83, 141)
	^*^J774a.1 macrophages (stimulated by ox-LDL to form foam cells)	VSMCs	VSMC adhesion and migration are promoted with the progression of cell phenotype switch and atherosclerosis	EVs can transfer integrins to VSMCs and promote the phosphorylation of ERK and Akt	(85)
	THP-1 macrophages (stimulated by Ang II)	HCAECs	Inflammatory factors are induced such as ICAM-1 and PAI-1 of HCAECs in a hypertensive state.	Reduced levels of miR-17 might contribute to the upregulation of ICAM-1 expression.	(86)
	^*^THP-1 macrophages (stimulated by UC)	ECs	The adhesion of human monocytes as well as ICAM-1 expression in ECs is augmented, promoting atherothrombosis	Moieties of superoxide and peroxides are exported on UC-stimulated MVs, which mediates endothelial activation	(88)
	THP-1 macrophages (unstimulated)	HMEC-1 cells	Cell migration of HMEC-1 cells is promoted, and angiogenesis might be improved	Exosomal miR-150 are delivered into HMEC-1 cells, effectively reducing c-Myb expression	(89)
T cells	Human T cells (activated by anti-CD3/CD28 Ab)	ECs	The endothelial permeability is increased, leading to acute cellular rejection in heart transplantation	High miR-142-3p enriched in exosomes can be transferred to ECs and downregulate RAB11FIP2 expression	(107)
	^*^Jukat T cells (unstimulated)	HUVECs	EC responses to VEGF and tube formation are modulated	CD47 expressed on EVs regulates intercellular communication	(108)
	^*^CEM T cells (stimulated by PHA, PMA and Act D)	HUVECs	EC apoptosis induced by Act D is prevented	MVs directly clear ROS during the early stage and increase manganese-superoxide dismutase afterward	(142)
Mast cells	HMC-1 Mast cell (stimulated by ITS)	HUVECs	PAI-1 secretion from ECs is increased and promotes a procoagulant state and endothelial dysfunction	Exosomes containing TNF-α precursor, angiotensinogen and factor V as well as prothrombin might activate PAI-1 expression	(125)

* *The results are mediated by other extracellular vesicles rather than exosomes*.

*BMDCs, bone marrow-derived dendritic cells; MI, myocardial infarction; LPS, lipopolysaccharide; ECs, endothelial cells; AngII, angiotensin type II; Ca/P, calcium and phosphate; MVs, microvesicles; ox-LDL, oxidized low-density lipoprotein; VSMCs, vascular smooth muscle cells; HCAECs, human coronary artery ECs; ICAM-1, intracellular adhesion molecule-1; PAI-1, plasminogen activator inhibitor-1; UC, unesterified cholesterol; Ab, antibody; HUVECs, human umbilical vein ECs; HMEC-1, human microvascular endothelial cell line; VEGF, vascular endothelial growth factor; EVs, extracellular vesicles; PHA, phytohemagglutinin; PMA, phorbol-12-myristate-13-acetate; Act D, actinomycin D; ROS, reactive oxygen species; ITS, insulin-transferrin-sodium selenite supplement; and TNF-α, tumor necrosis factor-α*.

Generally, exosomes derived from professional antigen-presenting DCs and macrophages are strongly implicated in myocardial infarction, atherosclerosis, certain cardiomyopathies, as well as endothelial dysfunction associated with hypertension (Table 3). These specific antigen-presenting vesicles have the advantage of a more controllable outcome over direct cell-contact approaches, which may cause unpredictable adverse events. As a result of the uptake of exosomes, subsequently activated lymphocytes and exosomes derived from them, especially immunosuppressive Tregs, may play a critical role. Although the potential cardiovascular impacts of exosomes from immune cells are only just beginning to be explored, along with insights in regenerative exosomes, they will advance our search for novel approaches to cardiovascular signaling and cardioprotection.

## Author Contributions

RW contributed to the article writing and designing. WG contributed to the article designing. KY and JG supervised and revised the article.

### Conflict of Interest Statement

The authors declare that the research was conducted in the absence of any commercial or financial relationships that could be construed as a potential conflict of interest.
